# Non-coding RNA-Mediated N6-Methyladenosine (m^6^A) deposition: A pivotal regulator of cancer, impacting key signaling pathways in carcinogenesis and therapy response

**DOI:** 10.1016/j.ncrna.2023.11.005

**Published:** 2023-11-13

**Authors:** Mehrdad Hashemi, Pouria Daneii, Mohammad Arad Zandieh, Rasoul Raesi, Neda Zahmatkesh, Mehrsa Bayat, Anwar Abuelrub, Zeinab Khazaei Koohpar, Amir Reza Aref, Ali Zarrabi, Mohsen Rashidi, Shokooh Salimimoghadam, Maliheh Entezari, Afshin Taheriazam, Ramin Khorrami

**Affiliations:** a Farhikhtegan Medical Convergence Sciences Research Center, Farhikhtegan Hospital Tehran Medical Sciences, Islamic Azad University, Tehran, Iran; b Department of Genetics, Faculty of Advanced Science and Technology, Tehran Medical Sciences, Islamic Azad University, Tehran, Iran; c Department of Food Hygiene and Quality Control, Division of Epidemiology, Faculty of Veterinary Medicine, University of Tehran, Tehran, Iran; d Department of Health Services Management, Mashhad University of Medical Sciences, Mashhad, Iran; e Department of Medical-Surgical Nursing, Mashhad University of Medical Sciences, Mashhad, Iran; f Department of Genetics, Zanjan Branch, Islamic Azad University, Zanjan, Iran; g Department of Health Sciences, Bahcesehir University, Istanbul, Turkey; h Neuroscience Laboratory, Health Sciences Institute, Bahcesehir University, Istanbul, Turkey; i Department of Cell and Molecular Biology, Faculty of Biological Sciences, Tonekabon Branch, Islamic Azad University, Tonekabon, Iran; j Belfer Center for Applied Cancer Science, Dana-Farber Cancer Institute, Harvard Medical School, Boston, MA, USA; k Department of Biomedical Engineering, Faculty of Engineering and Natural Sciences, Istinye University, Istanbul, 34396, Turkey; l Department Pharmacology, Faculty of Medicine, Mazandaran University of Medical Sciences, Sari, Iran; m The Health of Plant and Livestock Products Research Center, Mazandaran University of Medical Sciences, Sari, Iran; n Department of Biochemistry and Molecular Biology, Faculty of Veterinary Medicine, Shahid Chamran University of Ahvaz, Ahvaz, Iran; o Department of Orthopedics, Faculty of Medicine, Tehran Medical Sciences, Islamic Azad University, Tehran, Iran; p Department of Food Hygiene and Quality Control, Faculty of Veterinary Medicine, University of Tehran, Tehran, Iran

**Keywords:** m^6^A, Cancer, miRNA, lncRNA, circRNA, Immune response

## Abstract

The emergence of RNA modifications has recently been considered as critical post-transcriptional regulations which governed gene expression. *N*^6^-methyladenosine (m^6^A) modification is the most abundant type of RNA modification which is mediated by three distinct classes of proteins called m^6^A writers, readers, and erasers. Accumulating evidence has been made in understanding the role of m^6^A modification of non-coding RNAs (ncRNAs) in cancer. Importantly, aberrant expression of ncRNAs and m^6^A regulators has been elucidated in various cancers. As the key role of ncRNAs in regulation of cancer hallmarks is well accepted now, it could be accepted that m^6^A modification of ncRNAs could affect cancer progression. The present review intended to discuss the latest knowledge and importance of m^6^A epigenetic regulation of ncRNAs including mircoRNAs, long non-coding RNAs, and circular RNAs, and their interaction in the context of cancer. Moreover, the current insight into the underlying mechanisms of therapy resistance and also immune response and escape mediated by m^6^A regulators and ncRNAs are discussed.

## Graphical abstract

**Figure:**
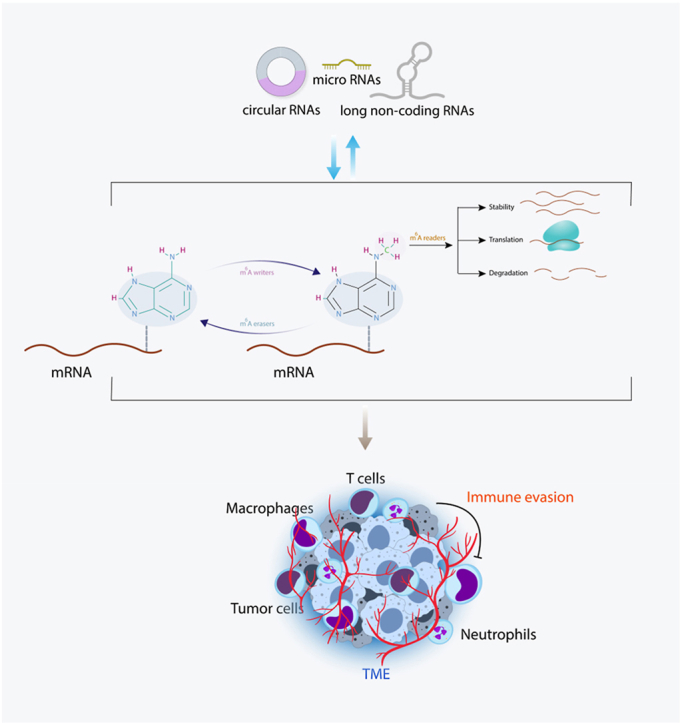

## Abbreviations

**ALKBH5**: Alpha-ketoglutarate-dependent dioxygenase homolog 5
**AML**: Acute myeloid leukemia
**ccRCC**: Clear cell renal cell carcinoma
**ceRNAs**: Competing endogenous RNAs
**CircRNAs**: Circular RNAs
**CTL**: Cytotoxic T lymphocytes
**CRC**: Colorectal cancer
**DLBCL**: Diffuse large B cell lymphoma
**EEC**: Endometrioid endometrial carcinoma
**eIF3**: Eukaryotic initiation factor 3
**EMT**: Epithelial-to-mesenchymal transition
**ESCC**: Esophageal squamous cell carcinoma
**Fe**^**2+**^: Ferrous iron
**FTO**: Fat mass and obesity-associated protein
**GAS5**: Growth arrest-specific 5
**GSC**: Glioblastoma stem-like cells
**HCC**: Hepatocellular carcinoma
**HNSCC**: Head and neck squamous cell carcinoma
**HPSCC**: Hypopharyngeal squamous cell carcinoma
**IGF2BPs**: Insulin-like growth factor 2 mRNA-binding proteins
**LncRNAs**: Long non-coding RNAs
**LSCC**: Laryngeal squamous cell carcinoma
**LUAD**: Lung adenocarcinoma
**m**^**6**^**A**: *N*^6^-methyladenosine
**METTL**: Methyltransferase-like protein
**MiRNAs**: Micro RNAs
**MTC**: m^6^A methyltransferase complex
**NcRNAs**: Non-coding RNAs
**NK**: Natural killer
**NPC**: Nasopharyngeal carcinoma
**NSCLC**: Non-small cell lung cancer
**OSCC**: Oral squamous cell carcinoma
**PD-1**: Programmed cell death protein 1
**PDAC**: Pancreatic ductal adenocarcinoma
**PiRNA**: Piwi-interacting RNA
**RBM15**: RNA-binding motif protein 15
**SiRNAs**: Small interference RNAs
**SncRNA**: Short ncRNA
**SnoRNAs**: Small nucleolar RNAs
**SnRNAs**: Small nuclear RNAs
**TAM**: Tumor associated macrophages
**TIME**: Tumor immune microenvironment
**TLS**: tertiary lymphoid structures
**TME**: Tumor microenvironment
**TNBC**: Triple negative breast cancer
**tRF**: Transfer RNA-derived RNA fragments
**tRNA**: Transfer RNA
**UTRs**: Untranslated regions
**WTAP**: Wilms' tumor 1-associated protein
**XIST**: X-inactive specific transcript
**YTH**: YT521-B homology
**YTHDC**: YTH domain-containing protein
**YTHDF**: YTH domain family protein
**Zc3h13**: Zinc finger CCCH domain-containing protein 13

## 1 Introduction

Gene expression and cell processes are tightly governed by genetic and epigenetic regulations which include mutation, chromosomal translocation, and modification changes of DNA and RNAs. Since the discovery of the first biological RNA in the 1960s, more than hundred types of modification changes to RNAs have been identified by now. *N*^6^-methyladenosine (m^6^A) modification has a dynamic, non-stoichiometric and reversible nature, and also is the most pervasive type of mRNA internal modification which is enriched in the 3ʹ untranslated regions (UTRs) close to the mRNA's stop codons [[1], [2], [3]]. With the help of immunoprecipitation and high-throughput sequencing, developing m^6^A-seq leads to providing the first transcriptome-wide profile of m^6^A in mammalian cells. Importantly, numerous m^6^A transcripts identified by these techniques corresponded to more than 7000 human genes. Of note, the studies carried out on m^6^A shaped the current picture of “epitranscriptome” [[4], [5], [6]]. M^6^A machinery complex is composed of proteins which act as “writer”, “reader”, and “eraser”. “M^6^A writers” mediate m^6^A modification, “readers” are recognized m^6^A sites to regulate downstream cell processes such as translation, and “erasers” reverse methylation [[7], [8], [9], [10], [11]]. Interestingly, m^6^A decorations have been revealed to play a critical role in various cancers [[12], [13], [14], [15]]. M^6^A modifications are involved in DNA damage response, fate of stem cells, RNA metabolism, gene translation, autophagy, and adipogenesis. In addition, they can be use as biomarkers of cancer occurrence [16]. Moreover, the importance of targeting m^6^A modification as a potential therapeutic approach to develop anticancer drugs is previously discussed [17].

It has been suggested for a long time that genomes’ sequencing is crucial for identifying the genes involved in cancer progression [18]. It is well-known now that genomic alterations are important in the carcinogenesis process, and reversing these alterations have been shown to be therapeutically effective as some inhibitors of epigenetic alteration are also approved by FDA [19]. In the recent decades, a plethora of non-coding RNAs (ncRNAs) such as micro RNAs (miRNAs), long non-coding RNAs (lncRNAs), and circular RNAs (circRNAs) are identified [[20], [21], [22], [23]]. According to their length, ncRNAs are divided into short ncRNAs, with less than 200 nt, and long ncRNAs, with more than 200 nt. miRNAs, piwi-interacting RNAs (piRNAs), small nucleolar RNAs (snoRNAs), small nuclear RNAs (snRNAs), and transfer RNA-derived RNA fragments (tRF) are among the small ncRNAs. These molecules work in cytoplasm and nucleus and controls a variety of vital functions such as transcription, translation, ribosome functions, etc [[24], [25], [26]]. NcRNAs are produced from the greater portion of the genome and do not code proteins but are the master regulators of gene expression and protein function. M^6^A deposition in ncRNAs has been reported to be enriched in primary miRNAs, (intergenic and intragenic primary miRNAs), in the whole body of the lncRNAs transcript. Dysregulation of ncRNAs has been proven by now to occur in all types of cancer, and this deregulation affects various cancer hallmarks. Interestingly, a number of RNA-based therapeutics is currently approved by FDA and a plethora of this type of therapeutics is in phase II or III clinical trials. More importantly, the immune system is yet a hurdle for RNA therapeutics [[27], [28], [29]].

Cancer systemically affects the immune system, and prolonged inflammation is considered as a cancer hallmark. It bears noting that a revolution has occurred in cancer therapy by targeting the immune system since the last decade. More importantly, every type of immune cell is involved in cancer biology. Also, the involvement of the immune system in cancer is multifaceted. For instance, hematopoiesis has been revealed to be disrupted in cancer, manifested by the presence of immature neutrophils and monocytes in the periphery and tumor microenvironment (TME). Moreover, immune cells show various phenotypes in different cancers. For example, in breast cancer, Natural killer (NK) cells have decreased expression level of activating receptors such as NKp30, and CD16, while the expression level of inhibitory receptor NKG2A is increased. Furthermore, in non-small cell lung cancer (NSCLC), the expression of activating receptors NCR1/2/3 in NK cells are decreased in peripheral NK cells [[30], [31], [32]]. Hence, devoting special attention to immunotherapy in cancer is of importance.

Regarding the critical role of m^6^A in carcinogenesis, this review intended to discuss the crosstalk between m^6^A modification regulators and ncRNAs in the context of various cancers. Besides, the current knowledge of the importance of interaction between m^6^A and ncRNAs effect on immune system response to tumor is discussed in this review.

## 2 N6-methyladenosine modification

M^6^A has been initially identified in 1974 in mRNA derived from Novikoff hepatoma cells [33], and is abundant in RNAs. M^6^A possesses chemical properties identical to adenine and it doesn't change the coding capacity of transcripts [34]. Discovery of m^6^A modification components has revealed that m^6^A is involved in the entire RNA life cycle, including mRNA nuclear translocation, molecular stability, localization in cell, splicing process of mRNA, and translation [35]. M^6^A modification is carried out by m^6^A methyltransferase complex (MTC) which called “m^6^A writer” and composed of methyltransferase-like protein 3 (METTL3) and 14 (METTL14) and their co factors including VIRMA (KIAA1429), Wilms' tumor 1-associated protein (WTAP), and RNA-binding motif protein 15 (RBM15) [1]. M^6^A methyltransferases were purified in HeLa cells for the first time in 1992, named MTA and MTB [36], and a subunit of MTA named METTL3 was discovered later in 1997 [37]. However, METTL14 was discovered 16 years later in 2013 and it has been revealed that METTL14 along with METTL3 form a stable complex for m^6^A deposition [38]. Another subunit of MTC is WTAP whose function has been proven in 2014. WTAP interacts with the METTL3-METTL14 complex to help them in localization into nuclear speckles and accelerates the catalytic function of them. It has been demonstrated that lack of WTAP is associated with reduction in METTL3 function [39]. Furthermore, studies revealed that two kinds of methylation exist based on the presence or lack of WTAP including WTAP-dependent sites which are static and correlate with mRNA stability, and WTAP-independent sites which are located at the first transcribed nucleotide. In this study, it was also found that KIAA1429 is crucial for the function of the MTC complex [9]. RBM15 role in m^6^A modification was revealed during a study on lncRNA X-inactive specific transcript (XIST), and the results have shown that RBM15 mediated MTC to specific sites in RNA [40]. Furthermore, Zinc finger CCCH domain-containing protein 13 (Zc3h13) has been revealed to be an important regulator of m^6^A methylation. In fact, Zc3h13 plays a critical role in anchoring the MTC in the nucleus [41]. Belonging to the family of alpha-ketoglutarate-dependent dioxygenase, Fat mass and obesity-associated protein (FTO) and Alpha-ketoglutarate-dependent dioxygenase homolog 5 (ALKBH5) were discovered as RNA demethylase of m^6^A in 2011 and 2013, respectively, which both mediate m^6^A demethylation in alpha-ketoglutarate- and Fe(II)-dependent manner [10,11,42].

**Fig. 1:**
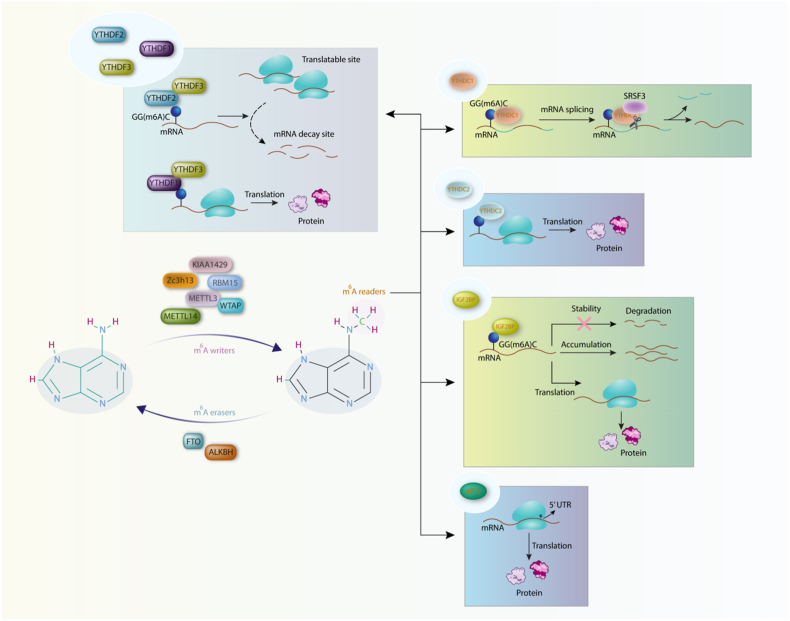
M^6^A writers, readers, and erasers, and their function in cell. M^6^A writers are responsible for their target methylation. The methylated sites are being demethylated by m^6^A erasers. M^6^A readers recognize methylated sites to mediate further cellular functions including translation, degradation, and stability of their target.

Also, a group of proteins which act as “m^6^A reader” are the YT521-B homology (YTH) domain family. YTH domain family protein 2 (YTHDF2), the first identified m^6^A reader, was found to target mostly mRNA, and also targets ncRNAs with G(m^6^A)C conserved core motif. In addition, YTHDF2 mediates mRNA traveling from the translatable pool to the sites of mRNA decay. For exerting this function, YTHDF2 uses its carboxy-terminal and amino-terminal domains which are responsible for binding to m^6^A-containing sites on mRNA and for the localization of the YTHDF2–mRNA complex to RNA decay sites [43]. YTHDF1 regulatory role was evaluated in a study by Wang et al. They have revealed that YTHDF1 recognizes m^6^A sites on mRNAs and augments ribosome occupancy of target mRNAs, thereby facilitating the initiation of translation [44]. In 2017, a study has elucidated that YTHDF3 is crucial for RNA-binding specificity of YTHDF1 and YTHDF2. Furthermore, YTHDF3 exerts its function in the early time point of RNA life cycle in cytosol compared to YTHDF1 and YTHDF2. Moreover, YTHDF3 modulates the turnover rate of m^6^A-modified transcripts [45]. YTH domain-containing protein 1 (YTHDC1) preferentially recognize the GG(m^6^A)C sequences [46] and modulate mRNA splicing process via recruiting and regulating pre-mRNA splicing factors to bind to their binding sites on target mRNAs. Indeed, YTHDC1 augments exon inclusion consistent with SRSF3 [47]. YTHDC2 is the last member of the YTH protein family whose function has been revealed in a study carried out on spermatogenesis. YTHDC2 selectively binds to the consensus motif of m^6^A and promotes the translation, and decreases mRNA abundance [48]. In addition to some of the YTH domain family of proteins which possess decay-promoting activity, a group of m^6^A readers have been identified recently which promotes the stability of their target mRNAs. Insulin-like growth factor 2 mRNA-binding proteins (IGF2BPs) including IGF2BP1, IGF2BP2 and IGF2BP3 target mRNA through their GG(m^6^A)C sequences. IGF2BPs regulate gene expression output at post-transcriptional level via enhancing the stability, accumulation, and translation of their target mRNAs (including Myc). They use their K homology domains for detection of m^6^A [49]. In addition, eukaryotic initiation factor 3 (eIF3) is another identified m^6^A reader which leads to direct recruiting of the 43S preinitiation complex to the 5′ UTR of mRNAs and subsequently stimulation of the translation initiation (Fig. 1) [8].

Importantly, the biological function and clinical significance of m6A modification have been revealed through various studies. In a study by Wang et al., the authors comprehensively discussed the critical function of ALKBH5 in human cancers including breast cancer, lung cancer, and gastrointestinal cancer, via targeting cancer cell proliferation, autophagy, apoptosis, invasion and metastasis [50]. In addition, another study was shown that mutations of FTO and YTHDF3 are associated with poor overall survival in LUAD [51]. M^6^A modifications also regulate cell processes such as RNA synthesis, splicing process, nuclear export, and mRNA translation and degradation in HNSCCs [52]. Furthermore, YTHDF2 is widely studied in a review, which mentioned the YTHDF2 implication in various aspects of human cancers through different mechanisms. The authors also discussed biological processes in cancer such as cell cycle, cell viability, proliferation, and metastasis. YTHDF2 also affect RNA metabolism through regulating mRNA decay and pre-ribosomal RNA processing. Importantly, YTHDF2 was found to be a promising prognostic biomarker in different cancers [52]. Moreover, Zhou and Gao revealed that m^6^A modification is highly related to the immune infiltration in early-stage LUAD [53]. As an example of metabolic reprograming regulation by m^6^A modifications, Wang and co-workers have shown that METTL3 promotes glycolysis and angiogenesis in gastric cancer. For exerting its function, METTL3 enhanced HDGF mRNA stability, then HDGF activates GLUT4 and ENO2 expression to increase glycolysis, accompanied by tumor growth and metastasis to liver [54]. Also, dysregulation of m^6^A regulators was reported to be related to therapy resistant and cancer immunity, and in drug-resistant cancer cells showed alterations in the levels of m^6^A regulators [55]. In uterine cancer, IGF2BP1 was found to be a pan-prognostic regulator of cancer, and can be used to predict the prognosis and clinicopathological characteristics [56]. In endocrine system tumors, WTAP and METTL16 expression was demonstrated to be positively correlate with overall survival [57].

## 3 Crosstalk between miRNAs and m^6^A regulators in cancer

Although more than 75 % of the human genome is transcribed to RNAs, only 2 % of them are translated to proteins [58]. More than 60 years have passed since the identification and recognizing the structure of the first short ncRNA (sncRNA), a transfer RNA (tRNA) [59]. In the 1990s, the first miRNA named Lineage defective-4 (lin-14) was recognized in *C.elegans* [60]. MiRNAs have ∼22 nucleotide in length and are generated by the function of RNA polymerase II (pol II) and a couple of enzymes and factors in the nucleus (DGCR8 and Drosha), and in the cytoplasm (Dicer). However, the origin of miRNA is different in non-canonical pathways. In fact, they could be derived from Mirtrons, shRNAs, and chimeric hairpins [[61], [62], [63], [64]]. MiRNAs are mainly bind to the 3ʹ UTR region of their targets. Nonetheless, some binding sites in promoter and 5ʹ UTR regions of targets have been reported for miRNAs [[65], [66], [67]]. MiRNAs mainly act as tumor promoting or suppressor factors. MiRNAs originated from genomic regions which amplified in cancer are oncogenic miRNAs, and those located in regions which undergo deletion in cancer function as tumor suppressor miRNAs [68]. The critical function of miRNAs in cell is widely studied during the past decades. MiRNAs are involved in numerous cellular processes including cell growth, cell death, and proliferation. They could target mRNAs for gene expression regulation, mediate translation inhibition, or provoke RNA degradation [69]. As an eminent characteristic, epigenetic alteration is seen in all types of cancers. Interestingly, it has been estimated that miRNAs regulate over one third of human genes [70]. MiRNAs are prone to epigenetic alteration and they also could regulate induction of epigenetic changes. For instance, miRNA-29 knocking down leads to upregulation of DNA methyltransferases and thereby inhibition of tumor suppressor factors [71].

**Fig. 2:**
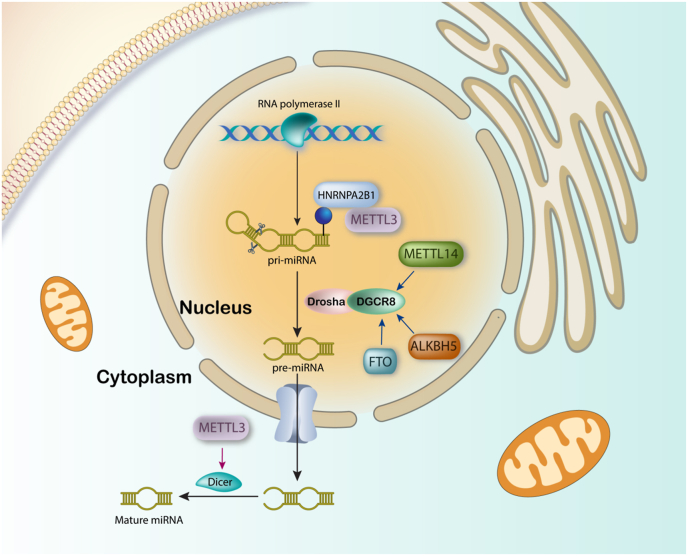
The process of miRNAs biogenesis and targeting sites of m^6^A regulators in this process.

Interestingly, the high accuracy of serum circulating m^6^A-miRNAs in cancer detection has been elucidated in a recent study, suitable for large-scale cancer screening as a non-invasive and cost-effective tool [72]. M^6^A marks on miRNAs play a critical role in miRNA processing through inhibition or promotion of this process [73]. In the case of miRNA biogenesis, METTL3 mediates m^6^A modification of pri-miRNAs to facilitate its recognition by DGCR8 [74]. In addition to DGCR8 [75], METTL3 is also able to affect the function of Dicer to promote miRNA processing in increasing angiogenesis and brain metastasis [76]. In addition, marking with m^6^A by METTL3 and reading these sites by HNRNPA2B1 has been elucidated to be of importance for further function of DGCR8 in miRNA biogenesis [77]. Interestingly, m^6^A modification of miRNAs is not always in favor of tumor progression. For example, METTL14 promotes m^6^A modification to enhance miRNA biogenesis in suppression of HCC tumor progression [78]. Furthermore, METTL14 also suppresses further process on pri-miRNA-126 by affecting DGCR8 to inhibit HCC cells metastatic capacity [78]. In contrast, it has been elucidated that METTL14 regulates m^6^A modification on hsa-miR-146a-5p to suppress its expression and increase breast cancer cells migration and invasion capacity [79]. Interestingly, it was revealed that ALKBH5 is the most potent member among all m^6^A regulators in esophageal cancer. It suppresses miRNA-194-2 biogenesis via demethylating pri-miRNA-194-2 in a m^6^A/DGCR8-dependent manner to decrease cancer malignancy [80]. FTO could also affect tumorigenesis by targeting miRNA biogenesis. FTO decreases miRNA-576 biogenesis by interacting with DGCR8 to increase expression level of CDK6 in promoting bladder cancer progression (Fig. 2) [81].

It is worth mentioning that besides affecting miRNA biogenesis for controlling tumorigenesis, other interactions between m^6^A regulators and miRNAs have been also revealed [82]. One of the regulatory functions of miRNAs in cancer is exerted via targeting m^6^A modification machinery. For instance, miRNA-600 suppresses lung cancer progression via targeting METTL3. Transfection of miRNA-600 was found to increase the Bax/Bcl-2 ratio, thereby triggering apoptosis through mitochondrial pathway. In addition, METTL3 activates PI3K/Akt and β-catenin pathways to promote tumor cell progression and survival rate. Furthermore, suppression of METTL3 leads to reduction of the DNMT3A m^6^A modification. Overall, miRNA-600 activates apoptosis and suppresses lung cancer cell progression via negatively affecting METTL3 [83]. In vitro, METTL3 has been revealed to be also targeted by miRNA-33a in NSCLC. MiRNA-33a directly targets the 3′ UTR of METTL3 mRNA to reduce its expression at both mRNA and protein levels, and negatively influences its downstream genes including EGFR, TAZ, MAPKAPK2 and DNMT3A. Suppression of METTL3 via miRNA-33a leads to decreased cancer cell proliferation [84]. In addition, METTL3 has been demonstrated to interact with SEC62 to trigger the m^6^A on SEC62 mRNA, and accelerate the stabilizing effect of IGF2BP1 on SEC62 mRNA. Intriguingly, miRNA-4429 inhibits METTL3 to suppress m^6^A-caused stabilization of SEC62 and impairs gastric cancer progression through this way [85]. In prostate cancer, it was revealed that KDM5A binds to the miRNA-495 promoter region to mediate its suppression by acting as an H3K4me3 demethylase enzyme. By inhibition of miRNA-495, its downstream target, YTHDF2, is overexpressed which induces MOB3B degradation by recognizing m^6^A sites on its mRNA, thereby promoting tumor progression [86]. In addition, miRNA-320d targets METTL3 to inhibit its further function on KIF3C and recognizes m^6^A sites by IGF2BP1 to suppress prostate cancer progression [87].

In a more complicated interaction, miRNA-135, as a tumor suppressor factor in breast cancer, inhibits ZNF217 to suppress EMT, invasion, and migration. ZNF217 prevents m^6^A modification of Nanog by METTL13 to increase its expression and function, followed by cancer progression. Thus, miRNA-135 has tumor inhibitory function in breast cancer through indirect regulation of m^6^A regulators [88]. In addition, miRNA-455-3p is also an ncRNA with tumor suppressor function which inhibits m^6^A modification mediated by METTL3 on its downstream target. It was found that when Wnt/β-catenin is activated in CRC cells, the host gene of miRNA-455-3p, COL27A1, is decreased and miRNA-455-3p undergoes downregulation. MiRNA-455-3p negatively regulates HSF1 mRNA and its suppression resulted in upregulation of HSF1, followed by its m^6^A modification mediated by METTL3. Then, HSF1 translation is increased which is further associated with tumor progression [89]. In addition, as a demethylase enzyme, FTO function has been shown to be prohibited by AMPKα2 in CRC. MiRNA-96 suppresses AMPKα2 to increase FTO expression, leading to deletion of m^6^A sites of Myc and subsequently its overexpression. This axis is in favor of tumor progression and apoptosis inhibition [90] (Table 1).

**Table 1:** Crosstalk between miRNAs and m^6^A regulators in cancer.

miRNA	Cancer type	m6A regulators	Signaling network	Function	Ref
MiRNA-30c-5p	Ovarian cancer	HNRNPA2B1	MiRNA-30c-5p/HNRNPA2B1/CDK19	Suppressed proliferation and metastasis.	[91]
MiRNA-148a-3p	Prostate cancer	METTL3	MiRNA-148a-3p/TXNIP	Enhanced proliferation, migration and invasion.	[92]
MiRNA-196b	CRC	METTL3	–	Enhanced migration and invasion.	[93]
MiRNA-92a	CRC	HNRNPA2B1	–	Noninvasive biomarker.	[94]
MiRNA-93-5p	Prostate cancer	HNRNPA2B1	MiRNA-93-5p/FRMD6	Enhanced proliferation and metastasis.	[95]
MiRNA-27b-3p	Glioma	METTL3	MiRNA-27b-3p/PDK1	Suppressed glycolysis and tumor progression.	[96]
MiRNA-93-5p	NSCLC	METTL14	MiRNA-93-5p/TXNIP	Enhanced proliferation, migration, and invasion.	[97]
MiRNA-139–5p	Prostate cancer	FTO	MiRNA-139–5p/ZNF217/PI3K/Akt/mTOR	Suppressed EMT and metastasis.	[98]
MiRNA-25-3p and miR-93-5p	Prostate cancer	HNRNPA2B1	CSNK1D/HNRNPA2B1/miRNA-93-5p/BMP and BAMBI/TGF-β CSNK1D/HNRNPA2B1/miRNA-25-3p/FOXO3	Enhanced proliferation and migration.	[99]

## 4 Crosstalk between circRNAs and m^6^A regulators in cancer

**Fig. 3:**
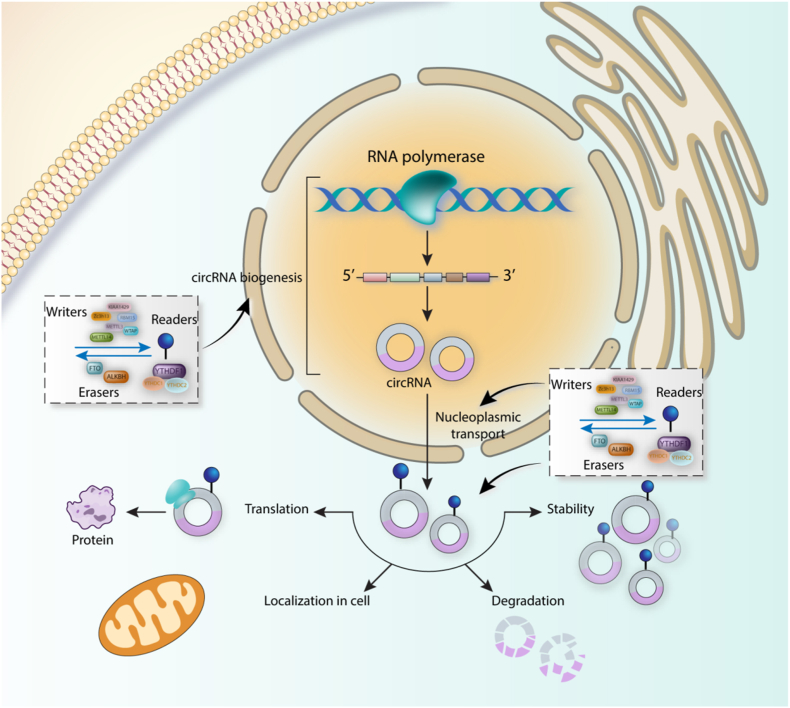
Regulatory role of m^6^A modifiers on circRNA. m^6^A regulators affect biogenesis of circRNA, their translocation from nucleus to cytoplasm, their stability, degradation, translation, as well localization in their functional sites in cells.

In addition to miRNAs, circRNAs could be controlled by the epigenetic modifications. Their closed-loop structure provides them to be more stable than the other linear ncRNAs transcript. Having nearly 30 years of history, circRNAs possess several critical functions in cells such as regulatation of transcription and translation, interacting with RNA-binding proteins, acting as miRNA sponges, and deriving pseudogenes. Similarly to miRNAs, aberrant expression of circRNAs has been seen in various cancers. They are generated from the back-splicing process or exon skipping of pre-mRNAs [100,101]. Interestingly, m^6^A modification in circRNAs has been elucidated to regulate circRNAs biogenesis, degradation, translation, stability, nucleoplasmic transport, and localization in the cell. Accumulating evidence has revealed the existence of m^6^A site on circRNAs. More importantly, m^6^A modification of circRNAs is involved in various aspects of immunity, pathogenesis of diseases, and also in the progression of different cancers (Fig. 3) [[102], [103], [104]]. Interestingly, m^6^A of circRNAs affect different aspects and is involved in all of the cancer hallmarks such as proliferation, invasion, metastasis, and resistance to chemotherapy and radiation therapy [105].

A recent study has revealed the interaction between m^6^A modification, circRNAs, and miRNAs in cancer. In this study, it was found that METTL14 expression is decreased in gastric cancer and its overexpression is associated with notable repression of growth and invasion of gastric cancer cells in vitro and in vivo. The authors reported that circORC5 is the downstream target of METTL14. Knocking down of METTL14 leads to reducing the m^6^A modification level of circORC5, and increases circORC5 expression. Furthermore, miRNA-30c-2-3p is recognized as a downstream target of circORC5. Upregulation of METTL14 is in favor of miRNA-30c-2-3p overexpression and subsequent decrease in its targets including AKT1S1 and EIF4B [106].

In cervical cancer, a study has revealed that circCCDC134 expression is increased and its overexpression facilitates proliferation and metastasis in vitro and in vivo. Furthermore, m^6^A modification of circCCDC134 tuned by ALKBH5 in an YTHDF2-dependent manner leads to its increased stability. In addition, circCCDC134 enhances HIF1A through interaction with p65. In another way, circCCDC134 serves as miRNA-503-5p sponge in cervical cancer to upregulate MYB, and subsequently triggering HIF1A transcription [107]. Interestingly, m^6^A modification of circRNAs has been elucidated in metabolic reprogramming of cancer. For instance, m^6^A-modified circFOXK2 was shown to be highly expressed in oral squamous cell carcinoma (SCC) and augmented malignant phenotypes. Indeed, circFOXK2 enhances glucose transporter 1 (GLUT1) stability via IGF2BP3 in a m^6^A-dependent manner, thereby promoting cancer cells aerobic glycolysis [108]. CircHPS5 has been shown to undergo m^6^A modification by METTL3 which increased its expression. YTHDC1 then recognizes m^6^A sites and accelerates circHPS5 translocation from nucleus to cytoplasm, where circHPS5 functions as a sponge for miRNA-370 to increase HMGA2 expression. This pathway ultimately leads to HCC cell tumorigenesis [109]. Similarly, m^6^A sites on circNSUN2 could be recognized by YTHDC1 to facilitate its cytoplasmic transport. In cytoplasm, circNSUN2 is targeted by IGF2BP2 to increase HMGA2 expression and stability, thereby enhancing CRC progression [110]. Furthermore, a study has reported another function for IGF2BP2. It was found that circARHGAP12, derived from ARHGAP12 mRNA, is translocated to cytoplasm by the function of IGF2BP2 following m^6^A modification. Then, the complex of m^6^A-modified circARHGAP12/IGF2BP2 targets FOXM1 mRNA to increase its protein level, thereby promoting cervical cancer progression [111]. In addition, circALG1 is generated from ALG1 pre-mRNAs through back splicing process. Interestingly, m^6^A modification of ALG1 pre-mRNAs by METTL3 resulted in the production of circRNAs with m^6^A sites. After its cytoplasmic export, circALG1 acts as competing endogenous RNAs (ceRNAs) for miRNA-342-5p to decrease its expression in an YTHDF1-dependent manner. Then, PGF and subsequently *p*-ERK are elevated which is in favor of CRC metastasis [112]. For inhibitory effects of m^6^A writers on oncogenic circRNAs, circPOLR2A is a good example. It has been reported that m^6^A-modified circPOLR2A is recognized and suppressed by YTHDF2 which leads to ccRC inhibition. However, circPOLR2A without m^6^A marks interacts with PEBP1 and UBE3C, resulting in ubiquitination and degradation of PEBP1, and subsequently activation of ERK signaling and ccRC progression [113]. Another example is m^6^A-modified circMDK which is recognized, stabilized and transported to cytoplasm by IGF2BP1 in HCC. In cytoplasm, circMDK act as a sponge for miRNA-346 and miRNA-874-3p to promote the expression level of ATG16L1, thereby augmenting proliferation, invasion, migration, while suppressing apoptosis. Intriguingly, it has been elucidated that delivering circMDK-siRNA by poly (β-amino esters) (PAEs) could significantly suppress circMDK oncogenic functions in cancer cells [114]. Also, circ3823 is an oncogenic ncRNA whoseexpression was demonstrated to be increased in CRC, associated with high proliferation, invasion, migration, as well angiogenesis. Circ3823 functions as a miRNA-30c-5p sponge to augment translation of its downstream target, TCF7, to accelerate tumor progression. The important point is that circ3823 degradation is regulated by m^6^A regulators including YTHDF2, YTHDF3, and ALKBH5 [115]. Moreover, it has been uncovered that m^6^A modification of circCPSF6 regulated by ALKBH5 and YTHDF2 is of importance for affecting HCC malignancy through targeting YAP1 [116].

**Fig. 4:**
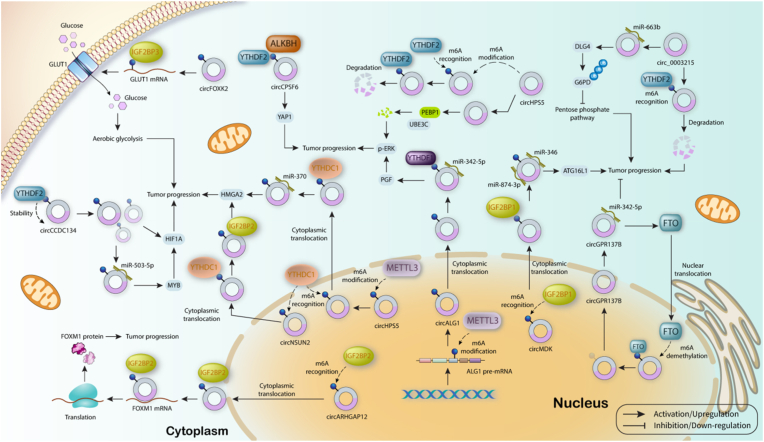
CircRNAs as downstream targets in cancer. M^6^A regulators which target circRNAs act as tumor suppressor or oncogenic factors in cancer. As tumor promoting factors, m^6^A regulators increase the stability of oncogenic circRNAs, promote circRNAs role on their downstream target, mediate their translocation to cytoplasm, and increase degradation of tumor suppressor circRNAs. On the contrary, m^6^A regulators with tumor suppressor function increase the stability of targeted circRNAs.

Interestingly, circGPR137B which has tumor suppressor function in HCC produces a positive feedback loop with FTO to suppress tumor cells proliferation, invasion, metastasis, and colony formation. For this purpose, circGPR137B suppresses miRNA-4739 to increase FTO expression, leading to translocation of FTO into nucleus to mediate demethylation of circGPR137B, and increase its stability and further functions [117]. Similarly, circ_0003215 is also a tumor suppressor circRNA with low expression level in CRC, and correlated with low TNM stage and lymph node metastasis. More importantly, this low expression is resulted from circ_0003215 degradation mediated by YTHDF2. In CRC, circ_0003215 increases DLG4 expression via inhibiting miRNA-663b. DLG4 suppresses the pentose phosphate pathway through augmenting the ubiquitination of G6PD, followed by cancer progression inhibition. However, degradation of circ_0003215 by YTHDF2 blocks this pathway and increases tumor progression through promoting metabolic reprogramming (Fig. 4) [118].

**Fig. 5:**
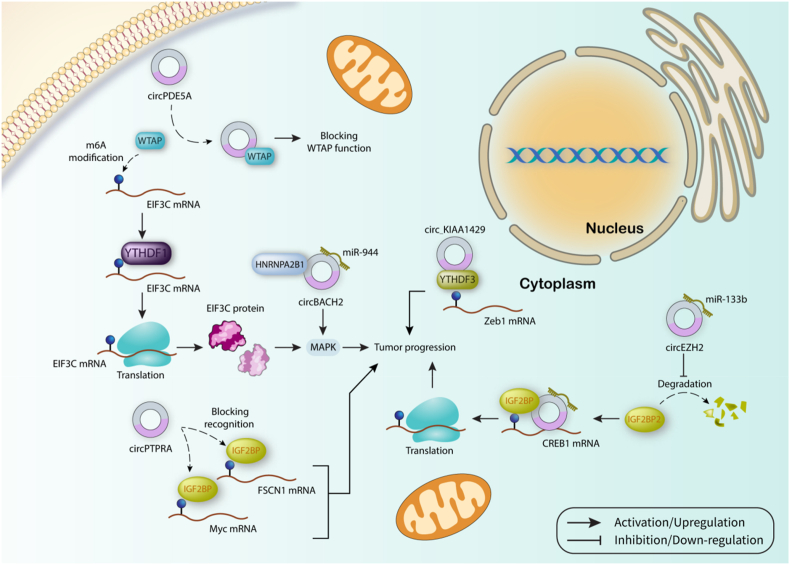
CircRNAs affecting m^6^A regulators. circRNAs affect m^6^A regulators function through inhibiting methylation process by writers, and preventing the recognition of downstream target and following processes including translation and degradation mediated by readers.

CircBACH2 overexpression has been elucidated to be in favor of breast cancer progression. Acting as has-miRNA-944 sponge, circBACH2 attenuates the inhibitory effect of has-miRNA-944 on HNRNPC, a highly expressed m^6^A regulator in breast cancer, thereby boosting breast cancer proliferation and progression through promoting phosphorylation of the MAPK signaling pathway [119]. Additionally, circ_KIAA1429 promotes the expression level of Zeb1 by increasing its mRNA stability in an m^6^A-YTHDF3 dependent manner to enhance HCC progression [120]. In addition, circEZH2 increases the expression level of IGF2BP2 and prevents its degradation via repressing miRNA-133b in CRC. Then, the complex of circEZH2/IGF2BP2 targets m^6^A-modified CREB1 to increase its protein level and downstream genes, thereby augmenting cancer progression [121]. Interestingly, circPTPRA is a tumor suppressor ncRNA in bladder cancer which interacts with KH domains of IGF2BP1 to endogenously block the recognition of IGF2BP1 to m^6^A-modified RNAs including FSCN1 and Myc [122]. CircPDE5A is derived from pre-PDE5A mRNA whose transcription is amplified by FOXO4. CircPDE5A attenuates prostate cancer progression by interacting with WTAP and preventing m^6^A modification of EIF3C and subsequent increase of its translation in an YTHDF1-dependent manner. Besides, EIF3C promotes MAPK signaling to augment metastatic capacity of cancer cells (Fig. 5) [123]. Table 2 provided some of the other circRNAs which are in interaction with m6A regulators in the context of cancer.

**Table 2:** Crosstalk between circRNAs and m^6^A regulators in cancer.

CircRNA	Cancer type	m6A regulators	Signaling network	Function	Ref
Hsa_circ_0058493	HCC	METTL3 and YTHDC1	Hsa_circ_0058493/YTHDC1	Enhanced tumor growth and metastasis.	[124]
Hsa_circ_0072309	NSCLC	FTO	Hsa_circ_0072309/miRNA-607/FTO	Enhanced proliferation, migration, and invasion.	[125]
Circ0000069	Cervical cancer	METTL3	Circ0000069/miRNA-4426	Enhanced proliferation and migration.	[126]
Circ1662	CRC	METTL3	Circ1662/YAP1/SMAD3	Enhanced invasion and metastasis.	[127]
CircMAP2K4	HCC	YTHDF1	CircMAP2K4/miRNA-139-5p/YTHDF1	Enhanced proliferation.	[128]
CircDLC1	HCC	KIAA1429	circDLC1/HuR/MMP1	Decreased proliferation and motility.	[129]
Circ-TNPO3	ccRCC	IGF2BP2	ESRP1/circ-TNPO3/IGF2BP2/SERPINH1/Snail-Slug	Decreased proliferation and migration.	[130]
CircMETTL3	TNBC	METTL3	CircMETTL3/miRNA-34c-3p/METTL3	Decreased proliferation, invasion, tumor growth and metastasis.	[131]
CircDLC1	Glioma	METTL3	CircDLC1/miRNA-671-5p/CTNNBIP1	Decreased malignant proliferation.	[132]
CircRNF13	Cervical cancer	METTL3and YTHDF2	CircRNF13/CXCL1	Enhanced the radio-sensitivity.	[133]
CircFOXK2	OSCC	IGF2BP3	CircFOXK2/IGF2BP3/GLUT1	Enhanced glycolysis.	[134]
CircAFF2	CRC	ALKBH5/YTHDF2	CircAFF2/CAND1/Cullin1/NEDD8	Enhanced the radio-sensitivity.	[135]
CircDDIT4	Prostate cancer	WTAP, METTL3, and METTL14	CircDDIT4/ELAVL1/HuR/ANO7	Decreased proliferation and metastasis, enhanced apoptosis.	[136]
CircRBM33	Prostate cancer	METTL3	CircRBM33/FMR1/PDHA1	Enhanced tumor growth, proliferation, invasion, aggressive phenotypes, mitochondrial respiration, and poor prognosis.	[137]
CircMYO1C	PDAC	METTL3 and IGF2BP2	CircMYO1C/PD-L1	Enhanced tumor immune surveillance.	[138]
CircASXL1	Ovarian cancer	METTL3 and IGF2BP1	CircASXL1/miRNA-320d/RACGAP1/PI3K/Akt	Decreased proliferation, invasion, and migration.	[139]
CircCDK1	LSCC	IGF2BP2	EIF4A3/circCDK1/IGF2BP2/CPPED1/PI3K/AKT	Enhanced tumor progression.	[140]
Circ-CCT3	HCC	ALKBH5 and METTL3	Circ-CCT3/miRNA-378a-3p/FLT-1	Enhanced proliferation, invasion, migration, and angiogenesis.	[141]
Circ_KIAA1429	HCC	METTL3	Circ_KIAA1429/miRNA-133a-3p/HMGA2	Decreased proliferation, and migration.	[142]
CircEPHB4	Glioma	MELLT3 and YTHDC1	CircEPHB4/SOX2/PHLDB2	Enhanced stemness and metastasis.	[143]
CircNFIX	Ovarian cancer	IGF2BP1	CircNFIX/miRNA-647/IL-6R/PD-L1	Enhanced proliferation, metastasis and immune escape.	[144]
CircRNF220	Osteosarcoma	METTL3	CircRNF220/miRNA-330-5p/survivin	Enhanced proliferation, invasion, and motility of osteosarcoma cells.	[145]
Circ-CTNNB1	Osteosarcoma	RBM15	Circ-CTNNB1/RBM15/HK2, GPI, and PGK1	Enhanced glycolysis, growth, invasion, and metastasis.	[146]
CircCMTM3	HCC	WTAP and IGF2BP1	CircCMTM3/IGF2BP1/PARK7	Enhanced tumor growth, and suppressed ferroptosis.	[147]
CircABCC4	Prostate cancer	METTL3 and IGF2BP2	CircABCC4/IGF2BP2/CCAR1/Wnt/β-catenin	Enhanced cell stemness, migration, and invasion.	[148]
CircSLC38A1	Bladder cancer	METTL3	CircSLC38A1/ILF3/TGF-β2	Enhanced invasion and metastasis.	[149]
CircUHRF2	CRC	METTL3 and IGF2BP1	CircUHRF2/IGF2BP1/DDX27	Enhanced stemness and metastasis.	[150]
Circ_0000390		METTL3	Circ_0000390/Notch1	Suppressed proliferation, migration, and invasion.	[151]
CircASPH	CRC	IGF2BP2	CircASPH/IGF2BP2/STING	Enhanced macrophage M2 polarization.	[152]
Circ_0001187	AML	METTL3	EIF4A3/Circ_0001187/miRNA-499a-5p/RNF113A/METTL3	Decreased proliferation and increased apoptosis.	[153]
CircFUT8	LUAD	YTHDF2	CircFUT8/miRNA-186-5p/FUT8	Enhanced malignant progression.	[154]

## 5 Crosstalk between lncRNAs and m^6^A regulators in cancer

XIST and H19 were the first lncRNAs which were discovered in the 1990s [155,156]. LncRNAs have more than 200 nucleotides and possess more spatial and temporal specificity compared to mRNAs [157,158]. LncRNAs are generated via RNA polymerase II and have a 5ʹ cap and 3ʹ polyadenylated tail. LncRNAs could be classified based on their length, function, biogenesis mechanism, which part of the genome they originate from, and where they localize in the cell. Based on their site of origin, lncRNAs could be named sense, antisense, intronic, and intergenic lncRNA. In addition, lncRNAs function in the nucleus, mitochondria or cytoplasm. Moreover, lncRNAs act as guide, scaffold, decoy, and ceRNAs in the cell [[159], [160], [161]]. The presence of m^6^A on lncRNAs has been seen to play roles in RNA-RNA and RNA-protein interaction, as well chromatin remodeling. Besides, antisense lncRNAs could regulate the abundance of m^6^A on the sense mRNA via recruiting m^6^A erasers [162]. Furthermore, m^6^A modification of lncRNAs could act as a structural switch, contributing with ceRNA function of lncRNAs, as well gene silencing [73].

Accumulating evidence has revealed the interaction between m^6^A modification and lncRNAs in the context of cancer. Interestingly, lncRNAs could be a target of m^6^A regulators, or even target them for their further functions. For instance, LNC942 elevates METTL14 mRNA stability and its m^6^A methylase activity, thereby increasing the mRNA stability of METTL14 downstream targets including CXCR4 and CYP1B1. Then, their expression is promoted which facilitates breast cancer tumorigenesis [163]. In colorectal cancer (CRC), a comprehensive analysis on m^6^A regulators has revealed that m^6^A-related lncRNAs demonstrated underlying mechanisms in CRC progression and tumorigenesis [164]. In a study, it was elucidated that lncRNA MIR100HG overexpression is in favor of enhanced epithelial-to-mesenchymal transition (EMT), invasion, metastasis, and maintaining cetuximab resistance in CRC both in vitro and in vivo. LncRNA MIR100HG recruits hnRNPA2B1 to maintainTCF7L2 mRNA stability, which is a major transcriptional co-activator of the Wnt/β-catenin signaling. Indeed, hnRNPA2B1 recognized the m^6^A site of TCF7L2 mRNA in the existence of lncRNA MIR100HG. As a reciprocal positive feedback loop, TCF7L2 augments lncRNA MIR100HG transcription in turn. More importantly, this axis has been revealed to be promoted in samples from CRC who have developed local or distant metastasis [165]. Another example is lncRNA STEAP3-AS1 which is a hypoxia-induced antisense lncRNA and is overexpressed in CRC. Under hypoxic conditions, HIF-1α activates lncRNA STEAP3-AS1 transcription to enhance proliferation and metastasis in vivo and in vitro. For this purpose, lncRNA STEAP3-AS1 interacts with YTHDF2 to mediate the dissociation of YTHDF2 with STEAP3 mRNA, thereby protecting STEAP3 from m^6^A modification-mediated degradation. Increased STEAP3 protein expression level is then followed by increased production of cellular ferrous iron (Fe^2+^). Accumulation of Fe^2+^ mediates the phosphorylation of GSK3β to suppress its kinase activity, thereby β-catenin is released and translocated into the nucleus to activate the Wnt signaling pathway. Altogether, this axis ultimately facilitates CRC progression [166]. Similarly, UCA1 is an lncRNA with oncogenic activity and high expression level in breast cancer. LncRNA UCA1 promotes proliferation and invasion, while suppresses apoptosis. For exerting its function, UCA1 augments DNA methylation of METTL14 to induce m^6^A modification of miRNA-375 and attenuate its expression. It has been shown that SOX12 is the downstream target of miRNA-375 and m^6^A modification of this miRNA resulted in increased SOX12 expression [167]. Interestingly, lncRNA LINRIS prevents K139 ubiquitination of IGF2BP2 to increase its stability and inhibit its degradation through the autophagy process in CRC. This axis ultimately augments Myc-mediated glycolysis [168].

In contrast to these examples, some lncRNAs serve as downstream targets for m^6^A regulators. M^6^A modification of lncRNAs is of importance for regulation of metabolic reprogramming in cancer. In vitro and in vivo, upregulation of lncRNA LINC00958 has been demonstrated to be associated with gastric cancer progression. M^6^A modification of LINC00958 mediated by KIAA1429 resulted in the increased GLUT1 mRNA stability in an m^6^A manner, thereby promoting the aerobic glycolysis [169]. In addition, lncRNA NDUFA4 mRNA is increased via m^6^A modification mediated by METTL3. This process leads to overexpression of NDUFA4 by IGF2BP1 function. NDUFA4 decreased ROS level while promoting MMP in gastric cancer. Further, NDUFA4 promotes glycolytic and oxidative metabolism in cancer cells to enhance their progression [170]. Interestingly, LINC00958 is capable of improving endometrial carcinoma progression via interaction with IGF2BP3, not regulating its expression. Furthermore, E2F3 expression is regulated by IGF2BP3 after generation of IGF2BP3- LINC00958 complex in favor of increasing endometrial cancer cells proliferation and migration [171]. Also, in an interesting study, the authors have revealed that LNCAROD overexpressed in head and neck squamous cell carcinoma (HNSCC) is associated with tumor progression, proliferation, migration, advanced T stage and poor overall survival. In this study, m^6^A modification of LNCAROD by METTL3 and METTL14 was found to be accompanied by increased LNCAROD stability. LNCAROD acts as a scaffold to suppress proteasome-mediated degradation of YBX1 through promoting YBX1-HSPA1A protein–protein interaction. This ternary complex ultimately leads to HNSCC progression [172]. Beside the METTL3 and METTL14, WTAP is also involved in m^6^A modification of lncRNAs. It has been uncovered that WTAP mediates m^6^A modification of lncRNA DIAPH1-AS1 and paves the way for the function of IGF2BP2 to increase the stability of lncRNA in nasopharyngeal carcinoma (NPC). Then, DIAPH1-AS1 promotes the formation of the MTDH-LASP1 complex in increasing cancer growth and tumorigenesis. In addition, WTAP upregulation in NPC is dependent on the function of KAT3A-mediated H3K27 acetylation [173]. In vitro, lncRNA NEAT1-1 upregulation promotes bone and long metastasis capacity of prostate cancer cells. It was elucidated that m^6^A sites on NEAT1-1 are crucial for the interaction between CYCLINL1 and CDK19 and augmentation of Pol II Ser2p level in the promoter region of RUNX2 [174]. In addition to m^6^A regulators, other upstream factors could modulate lncRNAs. For instance, SP1 regulates lncRNA THAP7-AS1 at transcriptional level to increase its expression, while METTL3 acts as m^6^A regulator in post-transcriptional level to promote its stability in an IGF2BP1-dependent manner, thereby increasing its function as an oncogenic lncRNA in gastric cancer. THAP7-AS1 interacts with CUL4B to increase its nuclear translocation, and also promotes the interaction between NLS and importin α1. CUL4B catalyzed H2AK119ub1 and EZH2 mediates H3K27me3 which leads to miRNA-223p and miRNA320a repression. Furthermore, PI3K/Akt signaling is activated, resulting in facilitation of gastric cancer progression [175]. Another m^6^A writer is METTL16 whose positive effect on cancer progression is well uncovered in a recently published study. It was elucidated that METTL16 promotes HCC proliferation, invasion, and metastasis, while suppresses apoptosis via m^6^A modification of lncRNA RAB11B-AS1. This modification is associated with decreased the stability of RAB11B-AS1, resulting in its downregulation [176]. Similarly, FENDRR is an onco-suppressor lncRNA which is regulated by YTHDF2 in endometrioid endometrial carcinoma (EEC) and is involved in cancer cell proliferation inhibition. YTHDF2 recognized the m^6^A sites on FENDRR, leading to its degradation and subsequent upregulation of SOX4, thereby promoting EEC cell progression [177].

The function of m^6^A regulators is varying based on the cancer type and also their downstream lncRNA target. For example, METTL14 mediates m^6^A modification of lncRNA XIST to facilitate its degradation mediated by YTHDF2 in CRC. XIST is an oncogenic lncRNA which promotes tumorigenesis and metastasis in CRC, but its functions are attenuated through m^6^A modification-mediated by METTL14 [178]. However, another study has indicated that m^6^A modification of lncDBET by METTL14 is important for its overexpression in breast cancer, and subsequently targeting PPAR signaling pathway to promote lipid metabolism via directly targeting FABP5 [179].

It bears noting that m^6^A modification could be also in contribution with tumor suppressor lncRNAs. As an example, overexpression of lncRNA growth arrest-specific 5 (GAS5) is associated with inhibition of CRC progression in vitro and in vivo. LncRNA GAS5 accelerates translocation of YAP from nucleus to the cytoplasm by interacting with its WW domain, followed by YAP phosphorylation, ubiquitination, and subsequently its degradation. Intriguingly, YTHDF3 is a downstream target of YAP which also mediates decay of m^6^A-modified lncRNA GAS5 as a negative functional loop [180].

In another aspect, demethylation of lncRNAs could be of importance in promoting cancer. As it was also mentioned before, ALKBH5 is a demethylase enzyme which deletes m^6^A sites on RNA transcripts. It could also mediate demethylation of lncRNA PVT1 in osteosarcoma to inhibit its YTHDF2-mediated degradation, thereby enhancing tumor proliferation and growth [181]. Similarly, demethylase activity of ALKBH5 on lncRNA RMRP has been also revealed to be associated with enhanced RMRP stability and subsequent promotion of proliferation, migration and invasion, and suppression of cell apoptosis in LUAD cells [182]. Furthermore, lncRNA NRON interacts with ALKBH5 to prevent Nanog m^6^A-mediated degradation, thereby promoting gastric cancer tumorigenesis [168]. The oncogenic lncRNA TRERNA1 whose expression is increased by the function of ALKBH5 in diffuse large B cell lymphoma (DLBCL), acts as scaffold to decrease the cyclin-dependent kinases inhibitor p21 through mediating H3K27me3 in its promoter region, by recruiting EZH2 [183]. Intriguingly, in glioblastoma stem-like cells (GSC), ALKBH5 promotes proliferation and tumorigenesis via targeting FOXM1 nascent transcripts and HuR plays a critical role in this interaction. More importantly, lncRNA FOXM1-AS, which is located in the nucleus, also facilitates this interaction. The lack of ALKBH5 and FOXM1-AS leads to suppression of FOXM1 pre-mRNA function via HuR, while their presence is accompanied by m^6^A sites deletion and high FOXM1 expression, thereby promotion of GSCs tumorigenesis [184]. In addition to ALKBH5, FTO could also affect cancer biology by targeting lncRNAs. In a study, it was revealed that FTO is critical for upregulation of lncRNA MALAT1 via mediating its m^6^A sites demethylation. Then, MALAT1 suppresses miRNA-384 to increase MAL2 expression, and subsequently promotes bladder cancer progression [185].

A different type of interaction between m^6^A regulators and lncRNAs could be seen in the study of Hou et al. They have elucidated that LINC00460 is highly expressed in CRC and its upregulation is in favor of cancer cell proliferation, EMT, invasion and migration capacity. Mechanistically, LINC00460 directly interacts with IGF2BP2 and DHX9 to augment the stability of HMGA1 mRNA. More importantly, m^6^A modification of HMGA1 mRNA by METTL3 is of importance for LINC00460/DHX9/IGF2BP2 complex binding and also for increasing its expression and further functions [186]. Similarly, lncRNA GATA3-AS interacts with KIAA1429 in the nucleus, leading to m^6^A modification of GATA3 mRNA and subsequently its downregulation, paving the way for liver cancer cell metastasis and tumor growth [187]. Interestingly, a study revealed that hypoxia-responsive lncRNA AC115619 encodes a micropeptide that impedes HCC progression by binding to WTAP and inhibiting m^6^A modification [188]. Table 3 provides a list of lncRNAs which have critical interaction with m^6^A modification machinery members in regulating cancer hallmarks.

**Table 3:** Crosstalk between lncRNAs and m^6^A regulators in cancer.

LncRNA	Cancer type	m6A regulators	Signaling network	Function	Ref
HNF1A-AS1	CRC	METTL3 and IGF2BP2	HNF1A-AS1/IGF2BP2/CCND1	Enhanced proliferation, migration, angiogenesis, and cell cycle progression. Reduced apoptosis.	[189]
MALAT1	Prostate cancer	METTL3	MALAT1/PI3K/Akt	Enhanced tumor cell growth and invasion	[190]
RP11	CRC	METTL3	RP11/hnRNPA2B1/Siah1-Fbxo45/zeb1	Enhanced migration, invasion, and EMT.	[191]
ABHD11-AS1	NSCLC	METTL3	ABHD11-AS1	Enhanced proliferation and Warburg effect.	[192]
PCAT6	Prostate cancer	METTL3 and IGF2BP2	PCAT6/IGF2BP2/IGF1R	Enhanced proliferation, migration, invasion, and bone metastasis.	[193]
THOR	–	METTL3 and YTHDF1- YTHDF2	–	Enhanced proliferation, migration, invasion, and colony formation.	[194]
LINC00958	Breast cancer	METTL3	LINC00958/miRNA-378a-3p/YY1	Enhanced tumor progression.	[195]
LCAT3	LUAD	METTL3	LCAT3/FUBP1/Myc	Enhanced proliferation, invasion, metastasis, survival rate, and cell cycle progression.	[196]
LIFR-AS1	Pancreatic cancer	METTL3	LIFR-AS1/miRNA-150-5p/VEGFA/PI3K/Akt	Enhanced tumor progression.	[197]
RMRP	NSCLC	METTL3	RMRP/YBX1/TGFBR1/SMAD2/SMAD3	Enhanced proliferation, EMT, migration, invasion, cancer stem cells characteristics, radio-resistance, and cisplatin resistance.	[198]
SPHK2	Gastric cancer	METTL3 and YTHDF1	SPHK2/KLF2	Enhanced proliferation, invasion, and metastasis.	[199]
DUXAP9	ccRCC	METTL3	DUXAP9/PI3K/Akt/GSK3β/Snail	Enhanced proliferation, EMT, motility. Reduced apoptosis.	[200]
MALAT1	IDH-wildtype glioma	METTL3	MALAT1/NF-κB	Enhanced malignant progression.	[201]
FAM225A	nasopharyngeal carcinoma	METTL3	FAM225A/miRNA-590-3p-miRNA-1275/ITGB3/FAK/PI3K/Akt	Enhanced proliferation, invasion, and metastasis.	[202]
UCA1	AML	METTL14	UCA1/METTL14/CYP1B1-CXCR4	Enhanced tumor progression.	[203]
LINC01320	Gastric cancer	METTL14	LINC01320/miRNA-495-5p/RAB19	Enhanced proliferation, migration, and invasion.	[204]
MALAT1	Thyroid cancer	IGF2BP2	MALAT1/miRNA-204/IGF2BP2/m6A-Myc	Enhanced proliferation, invasion, and metastasis.	[205]
LINC00901	PDAC	YTHDF1 and IGF2BP2	LINC00901/IGF2BP2/MYC	Enhanced growth and invasion.	[206]
GAS5	NSCLC	FTO	GAS5/UPF1/BRD4	Enhanced autophagic cell death.	[207]
SNHG1	CRC	METTL3	–	Enhanced proliferation and migration.	[208]
CACNA1G-AS1	Ovarian cancer	IGF2BP1	CACNA1G-AS1/IGF2BP1/FTH1	Suppressed ferroptosis and enhanced proliferation and migration.	[209]
SOX2OT	NSCLC	METTL3, METTL14, and IGF2BP2	SOX2OT/miRNA-186-5p/METTL3/14/IGF2BP2/GLI1	Enhanced cancer stemness.	[210]
LINC00839	Nasopharyngeal carcinoma	IGF2BP1	LINC00839/TAF15/AOC1	Enhanced tumor progression.	[211]
MALAT1	OSCC	METTL14	MALAT1/miRNA-224-5p/KDM2A	Enhanced tumor progression.	[212]
FAM83H-AS1	Gastric cancer	WTAP	–	Enhanced migration, proliferation, and invasion.	[213]
DGUOK-AS1	NSCLC	METTL3 and IGF2BP2	DGUOK-AS1/METTL3/IGF2BP2/TRPM7	Enhanced angiogenesis, proliferation and migration.	[214]
NEAT1	NSCLC	METTL3	NEAT1/miRNA-361-3p/HMGA1	Enhanced tumor progression.	[215]
SNHG3	Gastric cancer	METTL3	SNHG3/miRNA-186-5p/cyclinD2	Enhanced tumor progression.	[216]
MEG3	NSCLC	HNRNPA2B1	MEG3/miRNA-21-5p/PTEN/PI3K/AKT	Enhanced proliferation and invasion.	[217]
NNT-AS1	Pancreatic cancer	METTL3	HIF-1α/NNT-AS1/METTL3/HuR/ITGB1	Enhanced immune escape.	[218]

## 6 Crosstalk between piRNAs and m^6^A regulators in cancer

In addition to miRNAs, circRNAs, and lncRNAs, studies have revealed the interaction between m^6^A regulators and other ncRNAs such as piRNAs. PiRNAs are almost 30 nt in length, located in both nucleus, cytoplasm, and mitochondria. PiRNAs interact with the PIWI subfamily of Argonaute proteins and are derived from mRNA, transposons, or lncRNAs whose biogenesis is independent from Dicer [25,219,220]. Importantly, piRNAs are aberrantly expressed in various cancers and play critical roles in cancer hallmarks [221,222]. Interestingly, a number of studies also indicated the interaction between m^6^A regulators and piRNAs in cancer. For instance, Xie el al. have shown that piRNA-14633 targets METTL14 to promote cervical cancer cell proliferation, invasion and malignancy. PiRNA-14633 augments METTL14 mRNA stability and protein expression in cancer cells. Furthermore, piRNA-14633 increased m^6^A modification of CYP1B1 in a METTL14-dependent manner [223]. Likewise, piRNA-30473 has oncogenic function in DLBCL and augments aggressive phenotype, proliferation and cell progression. For exerting its function, piRNA-30473 increases WTAP, leading to upregulation of HK2 in an m^6^A-dependent manner in increasing DLBCL tumorigenesis. Besides, it was elucidated that piRNA-30473 expression is significantly correlated with poor prognosis [224,225]. In addition, piRNA-31106 was shown to be highly expressed in breast cancer and its upregulation is associated with cancer cell viability, invasion, and migration, while prevents apoptosis. Also, piRNA-31106 enhances the m^6^A modification and accelerates the expression of METTL3 to promote cancer progression [226]. Similarly, piRNA-17458 was shown to be upregulated in cervical cancer, and it mediates tumor progression by promoting m^6^A modification in a WTAP-mediated manner [227].

## 7 Role of m^6^A deposition in ncRNAs in regulation of immune system response in cancer

Cancer therapy has seen a revolution after the emergence of antibody-based immunotherapies from the past decade. It has been suggested that distinct types of tumor immune microenvironment (TIME) exist. Infiltrated-excluded TIME is characterized by the lack of cytotoxic T lymphocytes (CTL) in the tumor core and it is hypothesized that tumor-associated macrophages (TAMs) block the infiltration of CTLs into the tumor core. Besides, the expression level of markers by CTLs (such as IFNγ) is low in Infiltrated-excluded TIME. This type is seen in cancers such as CRC and PDAC. In contrast, in infiltrated-inflamed TIMEs programmed cell death protein 1 (PD-1) + CTLs are highly infiltrated into the tumor core, and PD-L1 is also highly expressed by tumor and myeloid cells. In infiltrated-tertiary lymphoid structures (TLS) type of TIME, immune cells (such as B cells, dendritic cells, and regulatory T cells) with a composition like lymph node are aggregated into the tumor site. Importantly, producing an immunosuppressive and protumoral environment are needed for tumors to support their progression and enhance immune escape [228]. It is worth noting that many of the m^6^A regulators could be involved in adaptive and innate immune evasion and could also modulate the chemokine expression through affecting the TIME. Besides, m^6^A modification plays critical roles in T cell maturation and differentiation, antigen-presenting to T cells, and regulation of immune checkpoints and chemokine [229,230]. Although the studies regarding the role of m^6^A regulators on immune cells are low, the existing evidence strongly confirmed that m^6^A modification machinery members play a critical role in immune cell response to tumors. For instance, YTHDF2 targets various factors such as STAT5 and Eemesodermin to maintain NK cell homeostasis, maturation, and anti-tumor immunity [231]. In addition, knock down of METTL3 in macrophages leads to inhibition of SPRED2 translation mediated by YTHDF1 which is further accompanied by upregulation of NF-kB and STAT3, and subsequent tumor progression. More importantly, loss of METTL3 attenuates the therapeutic efficacy of PD-1 checkpoint inhibitors [232]. Furthermore, m^6^A modification also plays a key role in reshaping the TME [233].

Interestingly, the correlation of m^6^A modification with ncRNAs in affecting tumor immunity is an emerging subject and remains to be studied well. However, some evidence has proven this correlation. For instance, m^6^A modification in small interference RNAs (siRNAs) has been shown in a study to be associated with innate immune evasion. In this study, IFN-β was used for evaluating the immune-response. It was demonstrated that the expression level of IFN-β was low following the introduction of m^6^A, representing decreased immune-response. In addition, m^6^A modification in the guide strand was accompanied by slight immune-response suppression. More importantly, m^6^A modification of siRNA leads to innate Immune evasion without decreased RNAi capacity of siRNA [234]. Accumulating evidence has shown that m^6^A modification of circRNAs could be a critical regulator in the innate immune response and tumor immunity. For instance, studies have shown that m^6^A modification of circRNAs results in innate immune system suppression. Following the m^6^A modification of circRNAs with METTL3, METTL14, and WTAP, m^6^A is recognized by YTHDF2 which leads to circRNAs degradation and subsequent attenuation of the innate immune system. In another word, m^6^A has been shown to be important in avoiding circRNA recognition by the immune system and marking it as “self”, which leads to innate immune surveillance mediated by YTHDF [235,236].

**Fig. 6:**
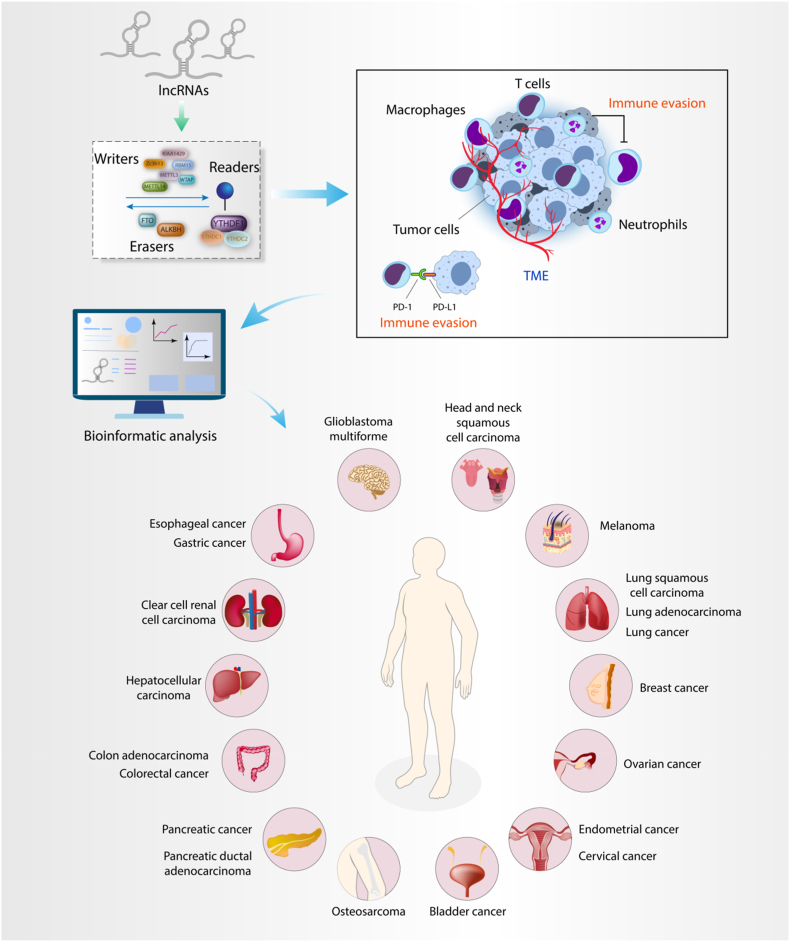
Cancer types in which m6A-related lncRNAs signature has been proven to be significantly correlated with immune cell infiltration, immunotherapy resistance and immune escape.

A study on m^6^A modification patterns of specimens from lung adenocarcinoma (LUAD) patients has revealed that m^6^A-related lncRNAs are notably consistent with immune-excluded, immune-inflamed and immune-desert phenotypes of tumors. Furthermore, based on an lncRNA score system, the authors have suggested that the high lncRNA score is associated with better overall survival, enhanced response to anti-PD1/L1 immunotherapy, and high sensitivity to erlotinib and axitinib chemotherapies [237]. Similarly, the study carried out by Li et al. has proved that a group of m^6^A-related lncRNA including LINC02362, lncRNA SNHG20 and lncRNA SNHG6 has high accuracy in prediction of overall survival of hepatocellular carcinoma (HCC). Besides, these lncRNAs are significantly related with TME and response to immunotherapy, and are also involved in immune-related pathways which play critical roles in HCC tumorigenesis [238]. In HCC, another research has focused on m^6^A-related lncRNA signature in two distinct subgroups. In a group of patients which the expression level of immune checkpoints CD47 and CD276 was high, they were more suitable for targeted therapy with immune checkpoint inhibitors. Overall, this study has indicated that the m^6^A-related lncRNA signature shows noticeable patterns in prognosis, TME characteristics, and immune properties of HCC patients [239]. Likewise, a study on bladder cancer has revealed an m^6^A-related lncRNA signature that was correlated with poor prognosis and suboptimal immune response. In addition, the authors have suggested that m^6^A modifications of lncRNA might contribute in the TME shaping and the modulation of tumor immune evasion, leading to the reduced responses to immunotherapy [240]. Wang et al. have performed a study on m^6^A-related lncRNA signature in gastric cancer and found that lncRNA signature is a promising source for predicting overall survival of gastric cancer patients without the necessity of considering other clinical variables. In this study, it was also suggested that some of these lncRNAs are related to response to the immune checkpoint inhibitor immunotherapy, thus lncRNA signature is of importance in developing personalized immunotherapies in gastric cancer [241]. Moreover, other studies also revealed m^6^A-related lncRNA signature which has reflected several types of immune cells infiltration in gastric cancer [[242], [243], [244]]. This association is also evaluated in other cancer types including CRC [245,246], ovarian cancer [247,248], endometrial cancer [249], HCC [250], cervical cancer [251,252], breast cancer [253], lung squamous cell carcinoma [[254], [255], [256]], HNSCC [257,258], pancreatic cancer [259,260], glioblastoma multiforme [261], melanoma [262], acute myeloid leukemia (AML) [263,264], esophageal cancer [265], osteosarcoma [266], and clear cell renal cell carcinoma (ccRCC) [267,268] (Fig. 6).

PD-1 is over activated by cancer cells to help them in escaping from the immune system. PD-1 signaling pathway disrupts T cell functions such as cytokine production and proliferation [269,270]. In addition, the aim of anti-PD-1/PD-L1 therapies is to prevent the interaction between PD-L1 located on the cell membrane of cancer cells and PD-1 located on the surface of T cells, thereby restoring the anti-tumor functions of T cells. Although a notable number of clinical trials have focused on anti-PD-1/PD-L1 therapy, some limitations still exist such as immune resistance, and overcoming this poor therapeutic outcome is of importance [271]. Interestingly, in a study the authors have revealed that there is a significant relation between the expression level of m^6^A regulators and HCC prognosis, and also immune microenvironment. They further elucidated that PD-L1 expression is low in a cluster with high expression level of m^6^A regulators associated with a good prognosis. In addition, miRNA-142-5p was found to be highly related to the most of the m^6^A regulators and its overexpression promotes the prognosis through affecting the tumor-infiltrating immune cells [272]. M^6^A-modification by METTL3 has been shown to mediate circIGF2BP3 overexpression and high stability in NSCLC in an YTHDC1-dependent manner. CircIGF2BP3 acts as miRNA-328-3p and miRNA-3173-5p sponge to suppress them and to increase PKP3 mRNA expression, and subsequently stabilizing OTUB1 in an FXR1-dependent manner. OTUB1 upregulation prevents PD-L1 degradation via removing ubiquitin chains, and ultimately increases resistance to anti-PD-1 immunotherapies and promotes immune evasion. Moreover, it was also found that circIGF2BP3 upregulation is associated with impaired CD8^+^ T cell infiltration and the aforementioned axis plays a critical role for CD8^+^ T cell response and infiltration [273]. In HCC, it was found that circRHBDD1 is overexpressed through EIF4A3-mediated exon back-splicing mechanism. CircRHBDD1 then recruits YTHDF1 to m^6^A-modified PIK3R mRNA to facilitate its translation. This process is in favor of increased aerobic glycolysis and promotes resistance to anti-PD-1 immunotherapies [274]. It bears noting that in HCC, lipopolysaccharide (LPS) of intestinal bacteria plays an important role in cancer progression. In HCC cells, LPS augments METTL14 upregulation which accelerates m^6^A modulation of lncRNA MIR155HG, leading to its overexpression via HuR. Then, MIR155HG serves as ceRNA for miRNA-223 to increase STAT1. This axis then causes an upregulation in PD-L1 expression which is mediated by STAT1, thereby increasing immune escape of HCC cells [275].

Various studies evaluated the interaction between m^6^A modification of ncRNAs and its impact on immune response in TME. The results of a research have shown that circNDUFB2 expression is decreased in NSCLC. In addition, circNDUFB2 suppresses growth and metastasis of NSCLC cells. For this purpose, circNDUFB2 acts as a scaffold to promote the interaction between TRIM25 and IGF2BPs, thereby facilitating ubiquitination and subsequently degradation of IGF2BPs. This ultimately resulted in augmentation of m^6^A modification of circNDUFB2. Furthermore, circNDUFB2 activates RIG-I-MAVS signaling cascades and recruits immune cells into the TME. Overall, circNDUFB2 regulates the degradation of IGF2BPs and elicits cellular immune responses to activate anti-tumor immunity in NSCLC. It bears noting that high immune response increased by circNDUFB2 is not dependent on m^6^A modification of circNDUFB2 [276]. In CRC, it was found that m6A-modified circQSOX1 function as sponge for miRNA-330-5p and miRNA-326 to augment PGAM1, which ultimately leads to immune escape via activation of glycolysis and inactivation of response against anti-cytotoxic T-lymphocyte-associated antigen-4 (CTLA-4) therapy [277].

In a pan-cancer analysis, it was found that UBE2C is up-regulated and its expression is correlated with cancer progression, poor prognosis, tumor stage, and lymph node metastasis. In addition, UBE2C has demonstrated to play a critical role in modulation of the immune response of human cancers. In this study, it was revealed that lncRNA SNHG1 stability is increased through m^6^A modification mediated by METTL3. Furthermore, lncRNA SNHG1 acts as miRNA-140-3p sponge to decrease its expression. Moreover, miRNA-140-3p targets 3ʹ UTR of UBE2C to negatively affect its expression. Thus, the METTL3/SNHG1/miRNA-140-3p axis ultimately results in the activation of UBE2C. More importantly, overexpression of UBE2C is associated with increased infiltration of diverse immune cells including CD8^+^ T cells, CD4^+^ T cells, neutrophils, dendritic cells, macrophages, and B cells. Besides, the results of analysis have revealed that high expression level of UBE2C is associated with immune checkpoint-related genes such as CD274, CTLA4, HAVCR2, LAG3, PDCD1, PDCD1LG2, SIGLEC15, and TIGIT [278]. Moreover, LncRNA PACERR expression is increased in TAM and its upregulation is accompanied by poor prognosis in pancreatic ductal adenocarcinoma (PDAC). Additionally, PACERR mediates M2 polarization of macrophages to facilitate tumor cell proliferation, invasion, and migration. To exert its functions, PACERR interacts with IGF2BP2 to promote KLF12 stability, and then increases the phosphorylated form of Akt through PTEN suppression. In addition, both of the PACERR-IGF2BP2 complex and activated form of Akt upregulates c-Myc. Besides, PACERR also suppresses miRNA-671-3p negative effect on KLF12 [279]. Despite the plethora of findings that have been made in the case of m^6^A modification, there is also a considerable gap in our knowledge about the importance of m^6^A modification in cancer immune response and evasion.

## 8 Role of m^6^A deposition in ncRNAs in regulation of therapy resistance in cancer

MiRNA-221-3p has been proven to possess an oncogenic role in breast cancer cells. METTL3 increases expression level of miRNA-221-3p by mediating m^6^A modification of its mRNA. MiRNA-221-3p negatively affects HIPK2 to increase Che-1, thereby increasing Adriamycin resistance. Interestingly, knocking down of METTL3 is in favor of drug sensitivity through reducing levels of miRNA-221-3p [280]. In contrast, miRNA-4443 affects METTL3 to increase drug resistance in NSCLC. MiRNA-4443 could be transferred from cisplatin-resistance cells to cisplatin-sensitive cells through exosomes in order to promote drug resistance in sensitive cells. For this purpose, miRNA-4443 regulates expression level of FSP1 in an m^6^A-dependent manner by modulating METTL3 function [281]. Intriguingly, as an upstream regulator of miRNA-493-5p, lncRNA MEG3 increases the expression level of this miRNA to suppress m^6^A modification of Myc mediated by METTL3, thereby increasing chemosensitivity to arabinocytosine by AML cells [282]. However, lncRNA MALAT1 targets miRNA-1914-3p to mediate resistance to cisplatin in NSCLC. MALAT1 whose expression is increased by METTL3/YTHDF3 complex acts as ceRNA for miRNA-1914-3p to suppress its inhibitory effect on YAP, thereby augmenting cisplatin resistance. In addition, METTL3 increases m^6^A sites on YAP to increase its translation in association with YTHDF3, YTHDF1, and eIF3b independent to MALAT1 and miRNA1914-3p [283]. Furthermore, miRNA-299-3p exerts oncogenic function in breast cancer via inhibiting WTAP expression. WTAP binds to m^6^A sites on lncRNA DLGAP1-AS1 to increase its stability and promote its function in increasing Adriamycin resistance. Interestingly, DLGAP1-AS1 sponges miRNA-299-3p to suppress its inhibitory function on WTAP in a feedback loop [284]. Another example is miRNA-584-5p which has tumor suppressor function in HCC. Similarly to the aforementioned examples, miRNA-584-5p is targeted by another lncRNA named DUXAP8. Acting as a sponge for miRNA-584-5p, DUXAP8 increases MAPK1 expression and subsequently MAPK/ERK pathway to promote tumor progression and also sorafenib resistance. More importantly, METTL3 is responsible for increasing DUXAP8 expression through promoting its m^6^A modification [285].

In glioma, exosomal circ_0072083 derived from temozolomide-resistant cells enhances drug resistance in sensitive cells. In sensitive cells, circ_0072083 suppresses miRNA-1252-5p to increase function of ALKBH5 in demethylation of Nanog, thereby increasing the drug resistance and tumor progression. It is worth mentioning that the results of this study have revealed that secretion of exosomes from resistant cells is accelerated by the Warburg effect [286]. Similarly, circ0008399 is upregulated in bladder cancer in an EIF4A3 dependent manner. In cancer cells, circ0008399 interacts with WTAP to recruit WTAP/METTL3/METTL14 complex in increasing TNFAIP3 mRNA stability in an m^6^A-dependent manner. Activation of this axis ultimately results in augmenting resistance of cancer cells to cisplatin therapy [287]. Likewise, circRNA-SORE is overexpressed and exerts oncogenic roles in HCC. CircRNA-SORE inhibits miRNA-103a-2-5p and miRNA-660-3p to activate the Wnt/β-catenin signaling pathway and promote sorafenib resistance. Interestingly, m^6^A modification of circRNA-SORE at a specific adenosine increases its stability in cancer cells [288]. In addition, circARHGAP29 expression level is increased in prostate cancer and its overexpression is associated with increased docetaxel resistance and aerobic glycolysis. EIF4A3 mediates cyclization and cytoplasmic transportation of circARHGAP29, where this circRNA augments LDHA mRNA stability through promoting its interaction with IGF2BP2 to increase glycolysis. Also, by interacting and stabilizing c-Myc mRNA and protein, circARHGAP29 facilitates LDHA transcription. Upregulation of LDHA by circARHGAP29 then leads to docetaxel-resistant prostate cancer cells [289]. Moreover, circVMP1 serves as a sponge for miRNA-524-5p to increase its downstream target, METTL3, and subsequent m^6^A modification of SOX2, thereby promoting cisplatin resistance in NSCLC cells [290]. In contrast, circASK1 plays as an onco-suppressor in LUAD and promotes gefitinib sensitivity. CircASK1 encoded ASK1-272a.a protein which is further required for ASK1/JNK/p38 signaling pathway. More importantly, endoribonucleolytic cleavage of m^6^A-modified circASK1 by YTHDF2 is responsible for low expression level of circASK1 in gefitinib-resistant cells [291]. Interestingly, it was found that overexpression of circCUX1 in hypopharyngeal squamous cell carcinoma (HPSCC) is accompanied by poor prognosis and resistance to radiotherapy. CircCUX1 is a downstream target for METTL3 to augment its m^6^A modification and increase its stability. Moreover, by binding to caspase 1 and suppressing its expression, circCUX1 reduces the release of inflammatory factors to promote radiotherapy tolerance in HPSCC [292]. One of the side effects of doxorubicin on nontargeted tissues is cardiotoxicity. Importantly, the role of circRNAs in doxorubicin-induced cardiotoxicity was studied, and it was revealed that circ-ZNF609 downregulation alleviates doxorubicin-induced cardiotoxicity by triggering apoptosis in cardiomyocytes. In addition, METTL14 was reported to regulate the function of circ-ZNF609, and FTO was found to be its downstream target [293]. Also, it was demonstrated that circPTEN regulates m^6^A modification of the PTEN promoter and GLUT1 expression to suppress ccRCC progression and increase the sensitivity of cancer cells to mTOR inhibitors [294]. Furthermore, it was found that circFBXW7 promotes resistance of LUAD and cancer stem cells to Osimertinib and prevents the renewal of stem cells. Interestingly, circFBXW7 was shown to be translated into short polypeptides named circFBXW7-185AA, which interacts with β-catenin in an m^6^A-dependent manner to increase its ubiquitination, thereby inactivation of Wnt signaling pathway. In addition, circFBXW7 promotes the sensitivity of cancer cells to tyrosine kinase inhibitors [295].

**Fig. 7:**
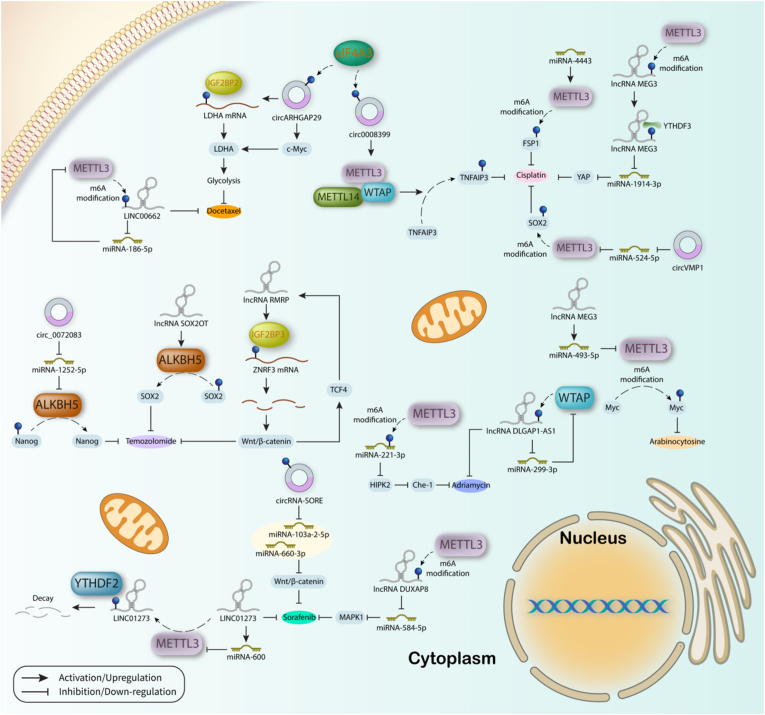
The interaction between ncRNAs and m^6^A regulators in mediating therapy resistance in cancer.

It was found that overexpression of lncRNA SOX2OT in glioblastoma is associated with tumor progression and temozolomide resistance in this cancer. SOX2OT modulates temozolomide resistance by promoting SOX2 expression and further activating the Wnt5a/β-catenin signaling pathway. Importantly, SOX2OT recruits ALKBH5 to regulate demethylation of the SOX2 transcript, which resulted in increased SOX2 expression and subsequently temozolomide resistance [296]. Furthermore, METTL3 increased LINC00662 stability to promote docetaxel resistance. Interestingly, LINC00662 suppresses miRNA-186-5p, and METTL3 is also a downstream target for miRNA-186-5p and this miRNA exerts its tumor suppressor functions by silencing METTL3 [297]. Intriguingly, LINC01273 is an oncogenic lncRNA which promotes miRNA-600 stability and also its function in METTL3 downregulation. Moreover, LINC01273 is a downstream target of METTL3 to undergo m^6^A modification followed by its decay in the presence of YTHDF2. This feedback regulatory axis has been elucidated to be involved in sorafenib resistance in HCC calls [298]. In addition, lncRNA RMRP augments temozolomide resistance in glioma by interacting with IGF2BP3 to decrease ZNRF3 expression and mRNA stability, as well reducing its interaction with Argonaute 2. By suppression of ZNRF3, RMRP increases Wnt/β-catenin signaling function. Interestingly, β-catenin enhances RMRP expression via targeting TCF4 in positive feedback loop [299]. Also, a study has shown that exosomal lncRNAs including LOC606724 and SNHG1 mediates drug resistance in multiple myeloma through interacting with METTL7A [300]. In addition, it was found that SRSF3, a critical regulator of mRNA alternative splicing process, promotes resistance to gemcitabine in pancreatic cancer cells through lncRNA ANRIL splicing process. More importantly, m^6^A modification of ANRIL mediated by METTL3 is crucial for this process. Interestingly, this process ultimately leads to formation of a DNA homologous recombination repair complex with Ring1b and EZH2 to prevent gemcitabine anti-cancer effect on cell (Fig. 7) [301]. Table 4 provides more examples for interaction between lncRNAs, m^6^A modification, and therapy resistance in cancer.

**Table 4:** Crosstalk between lncRNAs, m^6^A regulators, and therapy resistance in cancer.

LncRNA	Cancer type	m6A regulators	Drug	Signaling network	Ref
CEBPA-DT	OSCC	METTL3 METTL14	Cisplatin	CEBPA-DT/METTL3-METTL14/BHLHB9	[302]
CASC8	ESCC	ALKBH5 hnRNPL	Cisplatin	ALKBH5/CASC8/hnRNPL/Bcl2/caspase3	[303]
MALAT1	Breast cancer	METTL3	Adriamycin	METTL3/MALAT1/E2F1/AGR2	[304]
RHPN1-AS1	Ovarian cancer	METTL3	Cisplatin	METTL3/RHPN1-AS1/PI3K/AKT	[305]
NIFK-AS1	HCC	METTL3	Sorafenib	METTL3/NIFK-AS1/miRNA-637/AKT1	[306]
JPX	Glioblastoma multiforme	FTO	Temozolomide	JPX/FTO/PDK1	[307]
SNHG17	LUAD	METTL3	Gefitinib	METTL3/SNHG17/EZH2/LATS2	[308]
LINC00969	Lung cancer	METTL3 and YTHDF2	Tyrosine kinase inhibitor	LINC00969/METTL3/YTHDF2/NLRP3/caspase-1/GSDMD	[309]

## 9 Conclusions and outlook

M^6^A is the most abundant epigenetic alteration which regulates gene expression at post-transcriptional level. M^6^A is regulated by proteins that are known as m^6^A “writers”, which mediate RNA methylation, m^6^A “erasers”, which reverses the methylation, and m^6^A “readers”, which recognize m^6^A sites and determine RNA fate. Also, m^6^A modification has emerged as critical regulators of ncRNAs in cancer. M^6^A modifications have been found to intricately modulate key signaling pathways, adding another complexity to their involvement in cancer. These modifications can activate or suppress critical signaling cascades, ultimately influencing the tumor's behavior.

The most important point is that m^6^A regulators possess various functions likely based on the cancer type and their downstream target. Indeed, an m^6^A regulator with tumor suppressor function in a cancer type could exert oncogenic function in another one. For instance, YTHDF3 has the dual role of oncogenic and tumor suppressor in different cancers and future studies should uncover if an upstream mediator determines the exact function of this m^6^A reader in a cell. In addition, an ncRNA could be targeted by several m^6^A readers in cancer. Besides, the interaction between m^6^A regulators and ncRNAs is complicated and both them target each other and their interactions do not follow a certain pathway in cancer, and this makes them a promising target for cancer therapy.

Furthermore, miRNA biogenesis is considerably targeted by m^6^A regulators. Nonetheless, some of the pathways and precursors (such as Mirtrons) in miRNAs biogenesis are not revealed if they are also a target of m^6^A regulators and further studies could address this issue. More importantly, as miRNAs are downstream targets of most of the other ncRNAs including circRNAs and lncRNAs, regulation of miRNAs biogenesis by m^6^A regulators also indirectly affects the function of other ncRNAs in cancer. In addition to miRNAs, it is imperative to acknowledge the m^6^A modification equally significant roles in lncRNAs and circRNAs. These ncRNA molecules, when subjected to m^6^A modification, contribute to the intricate regulatory network in cancer development and progression. The inclusion of lncRNAs and circRNAs into our review expands our understanding of the multifaceted interactions of m^6^A modifications in the ncRNA realm. By discussing the dynamic alterations of m^6^A on these molecules, we shed light on additional layers of regulatory complexity. Thus, this review may hold the promise of inspiring novel targeted therapies and diagnostic strategies, with the potential to revolutionize the field of oncology in the future.

In addition to conventional cancer therapeutics, immunotherapy is a novel and promising technique but existing problems are immune escape and immunotherapy resistance by cancer cells. In contrast to the other cancer hallmarks, the effect of ncRNAs-m^6^A regulator complex on the tumor immunity is still poorly understood. A body of studies evaluated the prognostic value of m^6^A-modified ncRNAs in various cancers, as well as their ability in triggering immune cells infiltration. Yet, more in-depth in vitro and in vivo studies are required to a better understanding of critical function of m^6^A-modified ncRNAs in the immune cells’ response in cancer. As the evidence regarding the regulatory function of ncRNAs-m^6^A regulator complex on the tumor immunogenicity is limited to the analytic reports, more in-depth exploration of this interaction will bring new insights into cancer immunotherapy.

It bears noting that while m^6^A modification has gained substantial attention in cancer research, it is essential to acknowledge the ongoing debates regarding its functions and the potential for overestimation. These controversies stem from varying observations and interpretations across different studies. It is worth noting that the complexities of m^6^A biology make it a dynamic and evolving field. Some studies propose that m^6^A modification can either promote or suppress cancer progression, depending on the context and the specific target genes. For instance, it was suggested that the exact role of FTO remains controversial in human cancers [310]. Zhang et al. reviewed the dual function of FTO in ccRCC and state that this protein has context-dependent tumor-suppressive or oncogenic roles in a variety of solid cancers. The authors mentioned that although six studies shown tumor-suppressive function of FTO, but another three studies suggest that FTO is oncogenic protein [311]. This functional duality underscores the need for a comprehensive understanding of the regulatory mechanisms. Further, although different functions are attributed to the YTHDF proteins, but the accurate function of YTHDF proteins is still controversial [312]. Also, the translation process could be positively or negatively regulated m^6^A modifications, and depends on the binding sites of m^6^A regulators on mRNA [313].

In addition, the impact of m^6^A modification on cancer signaling pathways can vary based on the cellular environment, tumor type, and specific m^6^A regulator proteins. These context-dependent effects add to the complexity of its role in cancer. Also, it's important to recognize the challenges in accurately mapping m^6^A sites and quantifying their abundance, which could lead to discrepancies in reported results. Technical limitations should be considered when interpreting data. Zaccara and colleagues comprehensively reviewed the m^6^A mapping and measuring techniques, and discuss the advantages and limitations of each method. Importantly, despite the pivotal advance in m^6^A mapping, the information on stoichiometry is not provided by these methods. However, Mazter-Seq technique was shown to be useful for studying the stoichiometry of m^6^A modifications [314].

Moreover, the interplay between m^6^A and other epigenetic modifications, such as DNA methylation and histone modifications, adds another layer of complexity to the regulation of cancer-related genes. In light of these controversies, this article aims to provide a comprehensive overview of the current state of knowledge, incorporating various perspectives and insights from the literature. It is crucial to approach m^6^A modification as a dynamic regulatory mechanism in cancer, the full scope of which is still being elucidated. While this field holds immense promise, acknowledging the debates and challenges allows for a more nuanced understanding of its role in cancer and its implications for therapy response.

## Ethics approval and consent to participate

Not applicable.

## Consent for publication

Not applicable.

## Availability of data and materials

Not applicable.

## Funding

Not applicable.

## CRediT authorship contribution statement

**Mehrdad Hashemi:** Conceptualization. **Pouria Daneii:** Writing – review & editing. **Mohammad Arad Zandieh:** Investigation, Visualization. **Rasoul Raesi:** Validation. **Neda Zahmatkesh:** Data curation, Writing – original draft. **Mehrsa Bayat:** Investigation, Visualization. **Anwar Abuelrub:** Conceptualization. **Zeinab Khazaei Koohpar:** Data curation, Writing – original draft. **Amir Reza Aref:** Validation. **Ali Zarrabi:** Writing – review & editing. **Mohsen Rashidi:** Supervision. **Shokooh Salimimoghadam:** Supervision. **Maliheh Entezari:** Supervision. **Afshin Taheriazam:** Supervision. **Ramin Khorrami:** Supervision, Validation, Writing – review & editing.

## Declaration of competing interest

The authors declare that they have no known competing financial interests or personal relationships that could have appeared to influence the work reported in this paper.
